# Neonatal healthcare-associated infections in Brazil: systematic review and meta-analysis

**DOI:** 10.1186/s13690-021-00611-6

**Published:** 2021-06-01

**Authors:** Felipe Teixeira de Mello Freitas, Anna Paula Bise Viegas, Gustavo Adolfo Sierra Romero

**Affiliations:** 1 Escola Superior de Ciências da Saúde, Fundação de Ensino e Pesquisa em Ciências da Saúde, Brasilia, Brazil; 2 Hospital Materno Infantil de Brasília, Secretaria de Estado de Saúde do Distrito Federal, Brasilia, Brazil; 3 Secretaria de Vigilância em Saúde, Secretaria de Estado de Saúde do Distrito Federal, Brasilia, Brazil; 4 Núcleo de Medicina Tropical, Faculdade de Medicina, Universidade de Brasília, Brasilia, Brazil

**Keywords:** Healthcare-associated infections, Neonatal sepsis, Meta-analysis, Brazil

## Abstract

**Background:**

Healthcare-associated infections (HAI) are important causes of neonatal morbidity and mortality in developing countries. We reviewed the incidence and the pathogens involved in HAI among infants admitted to neonatal intensive care units (NICU) in Brazil.

**Methods:**

A search was conducted in the MEDLINE, LILACS and SciELO databases from January 1995 to October 2019. Two authors scrutinized potential articles independently, after one author selected them from screening abstracts from every article flagged as related to neonatal HAI. Then, they were included in the review if they met our inclusion criteria. The studies were evaluated based on a quality score proposed by the authors, rated 0 to 1, with 1 point as the best quality rate. Pooled estimates and 95% confidence intervals (95% CI) for HAI cumulative incidence and incidence density were calculated, when the same denominators were available, using meta-analysis. A quality effect was applied to the models using the MetaXL software. Heterogeneity was assessed using *I*^2^ statistics and the Cochran’s Q test.

**Results:**

Of a total of 5596 citations identified, 15 studies met the inclusion criteria for this review, which comprised 24,408 patients and 312,744 patient-days. Quality of the studies varied between 0.36 and 1 according to the adopted score, and six (40.0%) studies presented a score of 1. Pooled HAI incidence was 36.1 (95% CI 22.8–50.7) infections and 26.3 (95% CI 18.4–35.0) infected patients per 100 patients. Pooled HAI incidence density was 23.5 (95% CI 16.3–33.9) per 1000 patient-days. Pooled incidence density rates of bloodstream infection and ventilator-associated pneumonia were 13.1 per 1000 catheter-days (95% CI 4.3–40.1) and 7.9 per 1000 ventilator-days (95% CI 1.1–55.5), respectively. A high degree of heterogeneity was observed in all models (*I*^2^ > 98% and Cochran’s Q test with *p* < 0.05). Coagulase-negative *Staphylococci* (32.1%), *Staphylococcus aureus* (13.8%) and *Klebsiella* spp. (12.4%) were the most prevalent causative bacterial pathogens.

**Conclusions:**

The findings show high incidence of neonatal HAI in Brazilian NICU; therefore, efforts to standardize the collection and notification of HAI are needed in order to strengthen surveillance in the country and implement preventive measures, routine assessment, and close monitoring of neonates.

## Background

Brazil reduced infant mortality from 47.1 deaths per 1000 live births in 1990 to 15.3 deaths per 1000 live births in 2011, and thus achieved the fourth United Nations Millennium Goal [1, 2]. Most of the improvement was concentrated on post-neonatal mortality, which reduced at a rate of 8.1% per year from 1990 to 2007, whereas neonatal mortality reduced in a slower pace, 3.2% per year in the same period [3]. Thus, the proportion of neonatal mortality to mortality under the age of one has relatively increased, increasing from 49% in 1990 to 71% in 2015 [2]. Brazil has developed a new agenda, focused on actions to prevent neonatal mortality, to accelerate the reduction in infant mortality rates to as low as 10 deaths per 1000 live births by 2030, according to United Nations Sustainable Development Goals [4].

According to the national mortality system, the main conditions responsible for most of neonatal deaths in Brazil are complications associated with premature birth and low-birth weight, congenital anomalies, perinatal asphyxia, and infection [2]. Neonatal sepsis is one of the main causes of hospitalization and death in neonatal intensive care units (NICU) [5], and is considered healthcare-associated when infections are related to the care provided to pregnant women and neonates. There are two patterns of disease: early-onset neonatal sepsis (EOS) and late-onset neonatal sepsis (LOS). EOS is variably defined as occurring within 48–72 h after birth, and is related to care provided to women during gestation and delivery. In these cases, microorganisms are of maternal origin, acquired hours or days before or during delivery from the birth canal, and neonates may have a history of prolonged rupture of membranes, preterm onset of labor, chorioamnionitis, and peripartum maternal fever [6]. LOS is defined as occurring during 4–90 days of life, and is caused by microorganisms acquired during medical care and related to prematurity and low birth weight, prolonged use of intravascular access, mechanical ventilation, total parenteral nutrition, and exposure to broad spectrum antibiotics [7].

A national survey of healthcare-associated infections (HAI) was performed in 1994, and estimated a prevalence of HAI of 14.4% in NICU [8]. That triggered infection control policies, which culminated with a federal law making infection control compulsory in every hospital in the country in 1997 [9]. The first surveillance system for HAI in Brazil was developed in 2004, by the State of São Paulo, covering its territory [10] and in 2011 a national system was developed [11]. The system is dependent of notifications from a variety of hospitals, with different levels of complexity and no critical appraisal of the reliability and quality of the information reported has been performed so far. Moreover, as 98% of births occur in the hospital setting in Brazil [12], and because of the expansion of neonatal intensive care in the country in the last decades, HAI may account for a large proportion of neonatal deaths attributable to infection. Therefore, we decided to conduct a comprehensive systematic review and meta-analysis study, aiming to assess the incidence of neonatal HAI and identify their causative pathogens and their antimicrobial resistance profile in Brazilian NICU. This knowledge is essential to promote better policies and implement strategies to reduce neonatal mortality in the country.

## Methods

### Search strategy and inclusion criteria

This systematic review and meta-analysis aimed to identify studies on the epidemiology of neonatal HAI in Brazil: the proportion of EOS and the incidence of LOS of hospital origin. There was interest in the main sites of infection and rates of bloodstream infection (BSI) related to central venous catheters (CVC) and ventilator associated pneumonia (VAP), as well as in searching for the main microorganisms isolated in relevant microbiological samples and their antimicrobial resistance profile.

The Preferred Reporting Items for Systematic Reviews and Meta-analyses (PRISMA) guidelines were followed to perform this systematic review and meta-analysis [13]. A search with no language restriction was conducted in the online Medical Literature Analysis and Retrieval System (MEDLINE) for reports published between January 1995 and October 2019. The following syntax was applied: (“Cross infection” [MeSH] OR “Hospital Infection” OR “Hospital Infections” OR “Nosocomial Infection” OR “Nosocomial Infections” OR “Healthcare associated infection” OR “Healthcare associated infections” OR “Healthcare-associated infection” OR “Healthcare-associated infections” OR “sepsis” OR “infection” OR “infections”) AND ((“Infant, newborn” [MeSH] OR “infant newborn” OR “newborn infant” OR “newborn infants” OR “neonate” OR “neonates” OR “newborn” OR “newborns” OR “neonatal” OR “infant, premature” OR “infant” OR “infants”) OR (“Intensive care units” [MeSH] OR “Intensive care unit” OR “Intensive care units” OR “Intensive care” OR “Care Unit, Intensive” OR “Unit, Intensive Care”) OR (“Intensive Care Units, Neonatal” [MeSH] OR “Newborn Intensive Care Unit” OR “Newborn Intensive Care Units” OR “Neonatal Intensive Care Unit” OR “Neonatal Intensive Care Units” OR “Neonatal Intensive Care” OR “Newborn Intensive Care” OR “NICU”)) AND (“Brazil” [MeSH] OR “Brazil” OR “Brazilian” OR “Brasil”). The same search strategy was applied to regional, the Latin America and Caribbean Health Sciences Literature (LILACS) and the Scientific Electronic Library Online (SciELO) databases.

Articles were flagged if the title indicated that the study was related to neonatal HAI. Then one author reviewed the abstracts of the flagged articles and obtained the full text of potentially relevant studies that contained either full or partial data for proportions of HAI and the microbiological cause of these infections. Finally, each selected article was scrutinized by two researchers independently, and evaluated if it met the following inclusion criteria: 1) be conducted in Brazil; 2) present a sample including infants admitted to NICU, or infants with any invasive device (CVC or mechanical ventilation), or premature infants, or infants with birth weight < 1500 g; 3) present a report of HAI incidence or prevalence rates. Possible divergences between researchers in their evaluation were resolved by consensus. The reference lists of all reviewed studies were also screened for further eligible publications. References in duplicate, studies reporting outbreaks, publications reporting the same data, and non-original studies were excluded from this review. In the case of multiple reports by the same authors or same unit with overlapping study dates, the most comprehensive report was included.

### Data extraction

Extracted data included: authors; year of publication; place where the study was conducted; period of study; type of publication; study design; sample size; type of patient population; type of surveyed infection; surveillance methods; definitions used for diagnosis; reported infection incidence data and corresponding denominators; microbiological isolates. Microbiological data were only considered suitable for assessment when the number of bacterial isolates was reported.

### Quality score

**Fig. 1:**
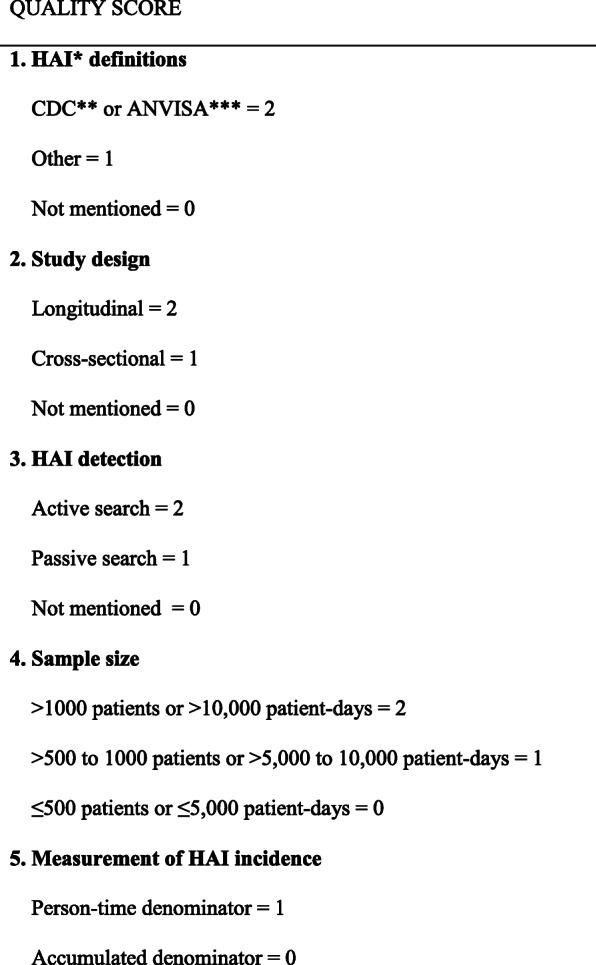
Quality score used to grade studies included in the systematic review. Final score ranged from 0 to 1, by dividing the total score by 9, the maximum possible score. *HAI* Healthcare-associated infections; *CDC* Centers for Disease Control and Prevention; *ANVISA* Agência Nacional de Vigilância Sanitária (Brazilian Sanitary Agency)

After in-depth review and data entry into a dedicated database, the selected studies were classified according to their quality based on a score created by the authors (Fig. 1). The score was based on five key areas of study methodology that may result in biases estimates: 1) use of HAI standardized definitions; 2) study design (cross-sectional or longitudinal); 3) method of HAI case detection (passive or active); 4) sample size; 5) measure of rates (person-time or cumulative incidence). The score for each area ranged from 0 to 2, except for the last area, which ranged from 0 to 1. In the end, the score was divided by 9, the maximum possible score, for standardization, and the final score ranged from 0 to 1, with 1 point as the best available quality.

### Statistical analysis

Data from incidence studies were pooled and the results in the same unit were summarized. The incidence of either infections or infected patients refers to the number of infection episodes or infected patients per 100 patients admitted to the NICU, respectively, during the study period. Infection incidence density refers to number of infection episodes per 1000 patient-days or device-days.

Median values and ranges of cumulative incidence and incidence density were reported. For studies reporting the same outcome measures and using the same methods, the data were pooled when the appropriate denominator was available. Pooled proportions and incidence densities were calculated using the MetaXL 5.3, a tool for meta-analysis in Microsoft Excel® [14]. Models were systematically applied with quality-effects estimator. Incidence and incidence density data were subjected to arcsine transformation to stabilize variance and prevent over or underestimation of study weights [15]. Heterogeneity was assessed using *I*^2^ statistics (values of 25, 50 and 75% represented low, medium and high heterogeneity, respectively) and the Cochran’s Q test when *p* < 0.05.

The total numbers of pathogens reported from BSIs and their antimicrobial resistance profile were descriptively presented.

## Results

**Fig. 2:**
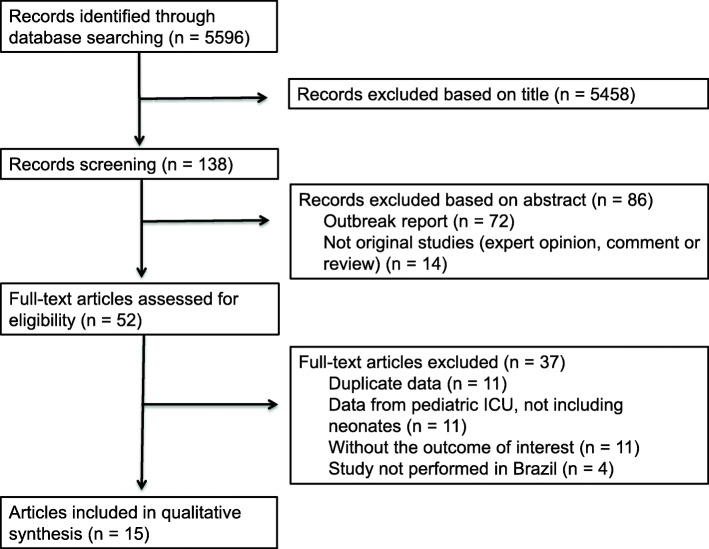
Flowchart showing the search and study selection strategy

Of a total of 5596 citations identified, 138 met the criteria for abstract review and 52 met the criteria for full-text assessment, of which 15 met the inclusion criteria for this review, namely, Kawagoe, JY et al. 2001 [16]; Nagata, E et al. 2002 [17]; Pessoa-Silva, CL et al. 2004 [18]; Couto, RC et al. 2007 [19]; Távora, AC et al. 2008 [20]; Pereira, SM et al. 2009 [21]; Catarino, CF et al. 2012 [22]; Dal-Bó, K et al. 2012 [23]; Freitas, BA et al. 2012 [24]; Ventura, CM et al. 2012 [25]; Romanelli, RM et al. 2013 [26]; da Silva, AR et al. 2013 [27]; de Souza Rugolo, LM et al. 2014 [28]; Urzedo, JE et al. 2014 [29]; Freitas, FTM et al. 2019 [30] (Fig. 2).

### Characteristics of the studies included in the review

The 15 studies comprised 24,408 patients (range from 203 to 6243; median of 866) and, although six studies did not report the number of patients-day, 312,744 patient-days (range from 1839 to 121,008; median of 14,256) were included in the remaining nine studies. Quality of the studies varied between 0.36 and 1.0 according to the adopted score, and six (40.0%) studies presented a score of 1.

The studies were conducted between 1993 and 2016 in 14 Brazilian cities located in the following regions of the country, 10 (66.7%), Southeast region; two (13.3%), South region; two (13.3%), Northeast region; one (6.7%), Mid-west region. No studies were conducted in the North region. Data were collected from 28 NICU, 14 (50.0%) of which were located in university hospitals, five (17.8%) in public hospitals, four (14.3%) in private hospitals, one (3.6%) in a philanthropic hospital, and four (14.3%) did not mention the type of hospital. Twelve studies (80.0%) included all infants admitted to the NICU, two (13.3%) included only infants < 1500 g, and one (6.7%) included only preterm infants. According to the definition of HAI, seven (46.7%) studies reported the use of the American Centers for Disease Control and Prevention (CDC) [27], four (26.6%) reported the use of the Brazilian Sanitary Agency (ANVISA) [28], two (13.3%) did not report which definition they used, one (6.7%) reported the use of both CDC and ANVISA definitions, and one (6.7%) developed its own definition. Table 1 shows a summary of the characteristics of the assessed studies.

**Table 1:** Characteristics of the assessed studies, neonatal healthcare-associated infections, Brazil

Authors/ Publication year	Study period	Study location	Hospital setting	Target population	HAI criteria	Number of patients	Number of patients-day	HAI incidence density*	BSI-CVC incidence density*	VAP incidence density*	Quality score
Kawagoe, JY et al. 2001	January 1993 to December 1997	São Paulo	Private	Every newborn admitted to NICU	CDC 1988	1544	12,266	23.8	NR	NR	1
Nagata, E et al. 2002	January 1999 to March 2000	Londrina	University	Every newborn admitted to NICU	CDC 1988	225	1839	62	59.9	142.8	0.81
Pessoa-Silva, CL et al. 2004	January 1997 to December 1998	Campinas, Rio de Janeiro, São Paulo	Private and university	Every newborn admitted to NICU	CDC 1988	4878	60,048	18.0	25.3	7.9	1
Couto, RC et al. 2007	January 1993 to December 2002	Belo Horizonte	Private and university	Every newborn admitted to NICU	CDC 1988	6243	121,008	29.8	13.7	4.3	1
Távora, AC et al. 2008	January 2003 to December 2003	Fortaleza	University	Every newborn admitted to NICU	CDC 1988	948	NR	NR	NR	NR	0.63
Pereira, SM et al. 2009	April 2001 to September 2004	Rio de Janeiro	Public	Every newborn < 1500 g	NR	203	NR	NR	NR	NR	0.45
Catarino, CF et al. 2012	January 2010 to December 2010	Rio de Janeiro	Public	Every newborn admitted to NICU	ANVISA	384	NR	NR	14.3	NR	0.63
Dal-Bó, K et al. 2012	January 2010 to December 2010	Tubarão	Philanthropic	Every newborn admitted to NICU	CDC 2008 and ANVISA	239	NR	NR	NR	NR	0.72
Freitas, BA et al. 2012	January 2008 to December 2010	Viçosa	Public	Every preterm newborn	ANVISA	267	NR	NR	NR	NR	0.54
Ventura, CM et al. 2012	March 2009 to August 2009	Recife	Public	Every newborn admitted to NICU	NR	218	2958	23.6	NR	NR	0.36
Romanelli, RM et al. 2013	January 2008 to December 2009	Belo Horizonte	University	Every newborn admitted to NICU	CDC 2008	886	14,256	22.7	18.1	5.7	1
da Silva, AR et al. 2013	January 2010 to June 2012	Rio de Janeiro	Private	Every newborn admitted to NICU	ANVISA	765	3051	18.7	NR	NR	0.72
de Souza Rugolo, LM et al. 2014	January 2006 to December 2008	Botucatu, Campinas, Porto Alegre, Ribeirão Preto, Rio de Janeiro, São Paulo	University	Every newborn < 1500 g	Own criteria	1507	NR	NR	NR	NR	0.81
Urzedo, JE et al. 2014	January 1997 to December 2012	Uberlândia	University	Every newborn admitted to NICU	CDC 2008	4615	62,412	17.6	17.3	3.2	1
Freitas, FTM et al. 2019	January 2014 to December 2016	Brasília	Public	Every newborn admitted to NICU	ANVISA	1506	34,906	20.4	18.6	NR	1

* Per 1000 patient-days or 1000 device-days

NR: Not reported

### Healthcare-associated infection incidence

Regarding HAI, four studies did not differentiate between early and late-onset sepsis, six studies only included LOS, and five studies differentiated EOS from LOS. Among these latter studies, the proportion of EOS ranged from 11.5 to 36.3%. Ten studies reported the site of infection, excluding EOS when this information was available. There were 7570 episodes of LOS among these studies: 3925 (51.8%) were BSIs, 877 (11.6%) conjunctivitis, 605 (8.0%) skin and soft tissue infections, 568 (7.5%) pneumonia, 301 (4.0%) ear, nose and mouth infections, 259 (3.4%) necrotizing enterocolitis, 174 (2.3%) urinary tract infections, 173 (2.3%) vascular infections, 157 (2.1%) meningitis, 131 (1.7%) surgical site infections, 117 (1.6%) omphalitis, and 283 (3.7%) other episodes.

There was heterogeneity regarding how studies reported HAI cumulative incidence. Among 13 studies, it was possible to use the overall number of infections, while among 11 studies, it was possible to use the number of infected patients, as numerator, per 100 admitted patients for the study period.

The median incidence of HAI was 34.2 (range from 7.4 to 64.9) infections per 100 patients and 25.7 (range from 13.8 to 46.6) infected patients per 100 patients. Pooled incidence of overall HAI was 36.1 (95% CI 22.8–50.7) infections per 100 patients and 26.3 (95% CI 18.4–35.0) infected patients per 100 patients. A high degree of heterogeneity was observed between these studies (*I*^2^ = 99.5%, *p* < 0.01 and *I*^2^ = 98.9%, *p* < 0.01, respectively).

**Fig. 3:**
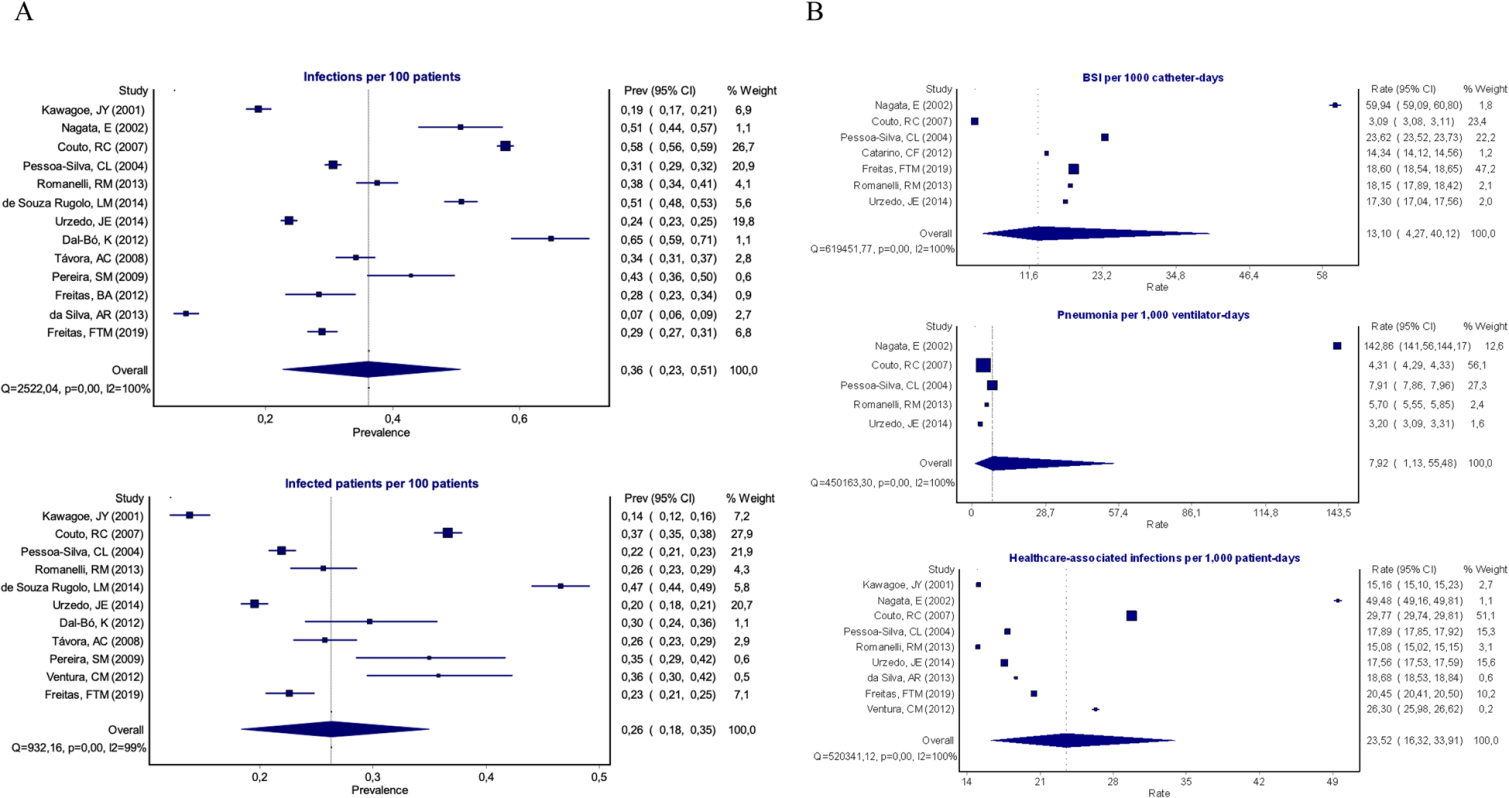
Pooled incidence and incidence density from HAI studies, Brazil, 1995–2014. A. Incidence of infections and of infected patients per 100 admitted patients. B. Bloodstream infection, pneumonia and healthcare-associated infection incidence per 1000 device-days or patient-days, respectively. *BSI* Bloodstream infection

HAI incidence densities were reported in nine studies, with a median of 23.6 (range from 15.1 to 62.0) infections per 1000 patient-days. Pooled HAI incidence density was 23.5 (95% CI 16.3–33.9) per 1000 patient-days. Seven studies reported BSI associated with CVC incidence density, with a median of 18.1 (range from 3.1 to 60.0) BSI per 1000 catheter-days. Pooled BSI-CVC incidence density was 13.1 per 1000 catheter-days (95% CI 4.3–40.1). Five studies reported VAP incidence density, with a median of 5.7 (range from 3.2 to 142.8) VAP per 1000 ventilator-days. Pooled VAP incidence density was 7.9 per 1000 ventilator-days (95% CI 1.1–55.5). A high degree of heterogeneity was detected in studies reporting HAI incidence density (*I*^2^ = 99.9%, *p* < 0.01), BSI-CVC incidence density (*I*^2^ = 99.9%, *p* < 0.01) and VAP incidence density (*I*^2^ = 99.9%, *p* < 0.01). Forest plots graphs summarizing the pooled HAI incidence rates are shown in Fig. 3.

### HAI pathogens and their antimicrobial resistance profile

Information on pathogens causing HAI was available from 12 studies. It resulted in 3803 bloodstream samples, and only one study differentiated EOS from LOS cultures. Gram-positive bacteria corresponded to 1944 (51.1%) of the pathogens, 1523 (40.1%) were gram-negative bacteria, and 336 (8.8%) were fungi. Coagulase-negative staphylococci (1275; 33.5%), *Staphylococcus aureus* (503; 13.2%), *Klebsiella* sp. (456; 12.0%), *Candida* sp. (227; 6.0%), and *Escherichia coli* (197; 5.2%) were the main pathogens identified. Table 2 shows a summary of the isolated pathogens.

**Table 2:** The pathogens responsible for HAI in NICU, isolated from bloodstream from 12 studies, Brazil

Pathogens	n (%)
Coagulase-negative *staphylococci*	1117 (32.1)
*Staphylococcus aureus*	482 (13.8)
*Klebsiella* sp.	431 (12.4)
Unspecified gram negative rods	293 (8.4)
*Escherichia coli*	192 (5.5)
*Candida* sp.	188 (5.3)
*Pseudomonas aeruginosa*	150 (4.3)
Unspecified fungi	120 (3.4)
*Enterobacter* sp.	115 (3.3)
*Acinetobacter* sp.	76 (2.2)
*Enterococcus* sp.	68 (1.9)
*Serratia marcescens*	61 (1.8)
*Proteus mirabilis*	48 (1.4)
*Streptococcus agalactiae*	46 (1.3)
Others	98 (2.8)
**TOTAL**	**3485 (100)**

The studies included were Kawagoe, JY et al.; Nagata, E et al.; Pessoa-Silva, CL et al.; Couto, RC et al.; Távora, AC et al.; Catarino, CF et al.; Dal-Bó, K et al.; Freitas, BA et al.; Romanelli, RM et al.; de Souza Rugolo, LM et al.; Urzedo, JE et al.; Freitas, FTM et al.

Only eight studies reported antimicrobial resistance. Methicillin resistant *S. aureus* (MRSA) was reported in five studies, and ranged from 1.2 to 28.3% among *S. aureus* isolates. Only one study reported one single sample of vancomycin resistant *Enterococcus* sp. *Enterobacteriaceae* resistance to 3rd-generation cephalosporin was reported in six studies, and ranged from 23.3 to 36.6% among *Enterobacteriaceae* isolates and was most common among *Klebsiella* spp. There were no reports of carbapenem resistant *Enterobacteriaceae* (CRE).

## Discussion

This systematic review and meta-analysis study showed that endemic HAI represents a major burden and a safety issue for infants admitted in Brazilian NICU. A country-level estimate of neonatal HAI is reported, and pooled estimates showed that HAI incidence is 26.3% and BSI incidence density is 13.1 per 1000 CVC-day. The Brazilian Sanitary Agency has developed a network for HAI surveillance since 2011 that made the notification of HAI compulsory for every hospital in the country. Such a large surveillance system in such a diverse country is prone to inaccuracy due to the large variety of health services and their levels of complexity and capability to perform high quality surveillance. To date, there is no publicly available evaluation of this surveillance system that can critically appraise its results. According to its first report, with data from 2011, the BSI rate in Brazilian NICU was 22.9 per 1000 catheter-days [11], and reached 7.5 per 1000 catheter-days in 2018 according to its last report, a decrease of 67.2%, but the 90th percentile was still 15.5 per 1000 catheter-days [31]. This figure is lower than the pooled rate observed in the present study; however, the comparison of actual rates with our estimates are inadequate because our study spans a long period, from the initial milestones of hospital infection control in Brazil: the first reported outbreaks in nurseries, the first national HAI survey and legislation in the mid-1990s to the present day. During this period, there were several changes in the HAI definitions for surveillance and many advances in their control, with infection rates gradually decreasing. The data shown here are similar to those from the International Nosocomial Infection Control Consortium (INICC), which involved 48 NICU from middle- and low-income countries (18 from Latin America) and showed a pooled mean of 16.37 BSI per 1000 CVC-days [32]. Nevertheless, the figures presented here are higher than the average incidence rates in developed countries. In American NICU, the HAI rates were 1.3 BSI per 1000 CVC-days and 0.9 VAP per 1000 ventilator-days [33]. In Germany, among very low birth weight infants, BSI incidence density rates were 8.6 per 1000 CVC-days and 2.7 VAP per 1000 ventilator-days [34].

Bloodstream was the most common site of infection, as reported elsewhere. Infants rarely manifest sepsis as a focal infection, which hinders the definition of a focal infection as pneumonia, for example. Possibly, the BSI rates are overestimated, and better definition of HAI in the neonatal period remains a challenge. Low prevalence of necrotizing enterocolitis, meningitis, and surgical site infection (SSI) was found. The possible explanation is that the surveillance definitions only consider necrotizing enterocolitis when there is a radiologic finding of intestinal wall suffering, excluding less severe cases. For meningitis, no study has reported the frequency of lumbar puncture, and if this is not an established practice in the NICU, many meningitis may go undetected, especially in very low birth weight infants. As for SSI, it was not reported whether the NICU performed surgery, as only NICU with high level of complexity perform neonatal surgery.

The microbiological data was not able to compare pathogens between EOS and LOS. The predominance of gram-positive cocci, such as coagulase-negative staphylococci (CoNS) and *S. aureus*, followed by gram-negative rods such as *Klebsiella* sp. or *Escherichia coli*, and a lower proportion of *Candida* sp., was the same profile reported by ANVISA [35] and reflects the LOS profile observed in developed countries [5, 33]. This profile, of predominantly hospital-acquired pathogens, indicates the adoption of complex tertiary level neonatal care with a high rate of invasive device use.

The frequency of antimicrobial resistance was observed is close to that reported by ANVISA. The main concern in Brazilian nurseries is extended-spectrum β-lactamase (ESBL) producing organisms. Over a third of the *Enterobacteriaceae* were resistant to cephalosporins, and there was a relevant proportion of MRSA [35]. This profile possibly reflects the powerful selective pressure of inappropriate and prolonged use of antimicrobial drugs, specially 3rd and 4th generation cephalosporins, such as cefotaxime and cefepime that favor the emergence of ESBL and *Candida* spp. in hospital nurseries. No reports of CRE were found. Emergence of CRE in Brazilian NICU seems rarer than observed in adult intensive care units.

Limitations to this study include that although we opted to have a broad search to increase its sensitivity, we had only a single author to review a large number of abstracts. We acknowledge that this may risk losing potential articles to the systematic review, thus the procedure was carefully done twice. There was absence of representation of large geographic areas of Brazil due to the simple lack of reported data. We believe that HAI incidence may be underestimated, because more organized services, which are capable of performing high quality surveillance with better results, usually report their data. Moreover, there was a large heterogeneity between the studies, especially regarding sample size and the criteria used to define HAI. Some studies used the American National Healthcare Safety Network (NHSN) criteria from the CDC [36], while others used the Brazilian criteria from ANVISA [37]. Both criteria are similar, but the ANVISA criteria is more specific for clinical BSI because, in the absence of a positive blood culture, it defines sepsis according to a score using white blood cell count and reactive C protein despite physician prescription of antibiotics, unlike the CDC criteria. To cope with heterogeneity between studies, we preferred to apply a quality-effects estimator for meta-analysis, because unlike random-effects model, quality-effects method allows to incorporate study quality assessments into the pooled estimates. Furthermore, the quality-effects model leads to the maintenance of the correct coverage probability of the confidence interval, regardless of the level of heterogeneity, as well as a lower observed variance compared to the random effects model [38, 39]. As there is no quality score for studies addressing neonatal HAI, we decided to create our own score based on objective parameters. This quality score was aimed at simplicity and ease of application and reproducibility by different researchers. Similar approaches using MetaXL quality-effects models have been used successfully in different studies [40]. Moreover, the quality-effects model is robust to subjectivity in quality assessment because it tolerates a fair amount of variability in reproducibility related to who assesses the quality of the studies. The reason is the way quality information is used; it does not require any information on the direction and magnitude of the bias induced by quality deficiencies. The credibility rank is the relative probability that the study is credible related to the best study in the meta-analysis, thus scale independent. Previous studies have demonstrated that despite a wide variation in the quality of input studies, the error and coverage of the estimator remain superior to the random-effects model [38, 39]. Thus, to improve neonatal infection research and promote transparency, clarity, and comparability of scientific reporting, urgent standards and criteria to report neonatal infection are needed to synthetize robust evidence to inform public health interventions. A recent extension of the Strengthening the Reporting of Observational Studies in Epidemiology (STROBE) statement has been published for Newborn Infection (STROBE- NI), which can be useful in the coming years [41].

## Conclusions

In conclusion, a high burden of HAI was observed among Brazilian studies, which affects the most vulnerable group of infants, usually preterm and low birth weight, or those with complex congenital anomalies who need intensive care. This fact highlights the urgent need to prevent preterm birth as a strategy to reduce neonatal mortality. This is especially important in Brazil, where preterm birth has increased over the past years, reaching 11.9% of all births [42], twice the percentage observed in developed countries (5.5%) [43]. Improving antenatal care, with special attention to poverty-related maternal conditions, such as infections during pregnancy, pre-eclampsia, gestational diabetes, vaginal bleeding, low body mass index, smoking, and alcohol or drug abuse would reduce preterm birth. Moreover, a decrease in the number of Caesarean sections would also contribute to reduce preterm birth in Brazil, where the rate of this type of delivery is one of the highest in the world (55%) [44]. Additionally, there is an enormous gap between HAI rates in developed and developing countries, where there are constraints in staff and shortage of supplies and resources [45]. In middle-income countries as Brazil, where most births occur in the hospital setting and tertiary neonatal care with high rate of invasive device use has become more accessible, national estimates of HAI incidence can assist with prioritizing and evaluating effectiveness control measures such as quality improvement methods aimed at HAI prevention, including hand hygiene compliance, BSI prevention bundles, safe surgery, and antimicrobial stewardship programs.

## Data Availability

Not applicable.

## Abbreviations

ANVISA: Agência Nacional de Vigilância Sanitária – Brazilian Sanitary Agency
BSI: Bloodstream infection
CDC: Centers for Disease Control and Prevention
CoNS: Coagulase-negative staphylococci
CRE: Carbapenem resistant *Enterobacteriaceae*
CVC: Central venous catheter
EOS: Early-onset neonatal sepsis
ESBL: Extended-spectrum β-lactamase
HAI: Healthcare-associated infection
INICC: International Nosocomial Infection Control Consortium
LILACS: Latin America and Caribbean Health Sciences Literature
LOS: Late-onset neonatal sepsis
MEDLINE: Medical Literature Analysis and Retrieval System
MRSA: Methicillin resistant *Staphylococcus aureus*
NHSN: National Healthcare Safety Network
NICU: Neonatal Intensive Care Unit
PRISMA: Preferred Reporting Items for Systematic Reviews and Meta-analyses
SSI: Surgical site infection
SciELO: Scientific Electronic Library Online
STROBE-NI: Strengthening the Reporting of Observational Studies in Epidemiology for Newborn Infection
VAP: Ventilator-associated pneumonia
